# Manuka honey enhanced sensitivity of HepG2, hepatocellular carcinoma cells, for Doxorubicin and induced apoptosis through inhibition of Wnt/β-catenin and ERK1/2

**DOI:** 10.1186/s40659-021-00339-1

**Published:** 2021-05-28

**Authors:** Heba R. Al Refaey, Al-Sayeda A. Newairy, Mayssaa M. Wahby, Chris Albanese, Mohamed Elkewedi, Muhammad Umer Choudhry, Ahmed S. Sultan

**Affiliations:** 1 Biochemistry Department, Faculty of Science, Alexandria University, Alexandria, Egypt; 2 Oncology and Radiology Departments, Lombardi Comprehensive Cancer Center, Georgetown University Medical Center, Washington, DC USA; 3 Department of Medical Laboratory Technology, Faculty of Applied Health Sciences Technology, Pharos University, Alexandria, Egypt; 4 Oncology Department, Lombardi Comprehensive Cancer Center, Georgetown University Medical Center, Washington, DC USA

**Keywords:** Manuka honey, Doxorubicin, Hepatocellular carcinoma cells, Apoptosis Induction

## Abstract

**Background:**

Recently, there is increasing awareness focused on the identification of naturally occurring anticancer agents derived from natural products. Manuka honey (MH) has been recognized for its biological properties as antimicrobial, antioxidant, and anticancer properties. However, its antiproliferative mechanism in hepatocellular carcinoma is not investigated. The current study focused mainly on investigating the molecular mechanism and synergistic effect of anticancer properties of MH on Doxorubicin (DOX)-mediated apoptotic cell death, using two different p53 statuses (HepG2 and Hep3B) and one non-tumorigenic immortalized liver cell line.

**Results:**

MH treatment showed a proliferative inhibitory effect on tested cells in a dose-dependent manner with IC_50_ concentration of (6.92 ± 0.005%) and (18.62 ± 0.07%) for HepG2 and Hep3B cells, respectively, and induced dramatic morphological changes of Hep-G2 cells, which considered as characteristics feature of apoptosis induction after 48 h of treatment. Our results showed that MH or combined treatments induced higher cytotoxicity in p53-wild type, HepG2, than in p53-null, Hep3B, cells. Cytotoxicity was not observed in normal liver cells. Furthermore, the synergistic effect of MH and Dox on apoptosis was evidenced by increased annexin-V-positive cells and Sub-G1 cells in both tested cell lines with a significant increase in the percentage of Hep-G2 cells at late apoptosis as confirmed by the flow cytometric analysis. Consistently, the proteolytic activities of caspase-3 and the degradation of poly (ADP-ribose) polymerase were also higher in the combined treatment which in turn accompanied by significant inhibitory effects of pERK1/2, mTOR, S6K, oncogenic β-catenin, and cyclin D1 after 48 h. In contrast, the MH or combined treatment-induced apoptosis was accompanied by significantly upregulated expression of proapoptotic Bax protein and downregulated expression of anti-apoptotic Bcl-2 protein after 48 h.

**Conclusions:**

Our data showed a synergistic inhibitory effect of MH on DOX-mediated apoptotic cell death in HCC cells. To our knowledge, the present study provides the first report on the anticancer activity of MH and its combined treatment with DOX on HCC cell lines, introducing MH as a promising natural and nontoxic anticancer compound.

## Background

Hepatocellular carcinoma (HCC) is one of the most malignant tumors emanating from hepatocytes. It is the highest prevalent primary liver cancer, representing the sixth most common cancer worldwide [1]. Moreover, HCC-related mortality ranks second globally, with a higher prevalence in males than females [1, 2]. In developing countries, particularly those in East Asia and sub-Saharan Africa, the incidence of HCC and the consequent mortality are significantly higher than in their developed counterparts [1]. Egypt has been facing a growing incidence of HCC, which represents the highest leading cause of local death among all other cancers [3]. This is mostly due to the high prevalence of endemic viral hepatitis, caused by hepatitis B (HBV) and hepatitis C viruses (HCV), compared to other risk factors [4, 5]. Both viruses are associated with the progression of a series of events, from chronic hepatitis to cirrhosis, then finally to HCC [1]. Since HCC has a very poor prognosis at the early stages, with rapid growth and a high rate of metastasis, most HCC patients are diagnosed at the advanced stages [6].

Currently, the conventional treatments for HCC, such as liver transplantation or resection [7], radiotherapy, and chemotherapy, have severe side effects. As drug resistance and adverse effects remain two critical hurdles [8], the necessity of beefing up the development of new anticancer agents has become apparent. For a substance to be utilized as a chemopreventive and chemotherapeutic agent in cancer treatment, it should exhibit high efficiency in hampering tumor growth, induce low side effects and alleviate the effect of carcinogenic agents [9]. Nowadays, significant awareness has been raised to identify naturally occurring anticancer agents derived from food and natural products. Among these natural products is honey, which has a potent anticancer effect, as it can suppress carcinogenesis by modulating or interfering with the molecular events of the initiation [10], proliferation [11], and progression stages [12].

Honey has a series of medicinal properties, such as anti-inflammatory [13], wound healing [14], anti-oxidative [15, 16], anti-diabetic [16, 17], antimicrobial [18], antibacterial [19, 20], antihyperlipidemic [21], antiproliferative, antimetastatic and antitumor properties [22]. These medicinal effects can be attributed to the various pharmacologically active constituents of honey, especially flavonoids and phenolic components [23, 24]. Therefore, kinds of honey possessing high phenolic and flavonoid content, such as Manuka Honey (MH) are deemed very medicinally attractive [24].

MH is monofloral honey obtained from the Manuka tree (*Leptospermum scoparium)*, belonging to the family Myrtaceae, and collected by honey bees called (*Apis Mellifera*) in New Zealand and the Eastern region of Australia [25]. Isolation and characterization of the bioactive fraction of MH by using HPLC demonstrated that it comprises a complex mixture of carbohydrates, fatty acids, proteins, vitamins, and minerals containing various kinds of phytochemicals rich in polyphenols and flavonoids, that have been identified with potent ROS scavenging activity [25–29]. The major flavonoids are pinobanksin and pinocembrin, representing approximately 36% and 23% of the total flavonoid content, respectively followed by the presence of quercetin (11.81%), luteolin (8.30%), kaempferol (3.70%), chrysin, and galangin. Leptosin derivatives and methyl syringate were characterized as the major compounds in MH, representing approximately 35.5% and 43.87% of the total phenolic content, respectively [30, 31]. Moreover, MH contains other various phenolic compounds, such as apigenin, isorhammentin, 4-hydroxybenzoic acid, and caffeic acid. This dark honey has recently garnered a lot of attention and consideration for its biological properties, especially its antimicrobial effects, antioxidant efficacy, and potential role in wound healing [27, 32].

The current study sheds light on MH and its concurrent administration with Doxorubicin (DOX) as a potential anti-cancer agent. It has been priorly demonstrated that MH (Unique Manuka factor, UMF10 +) inhibited cell proliferation in murine melanoma, colorectal carcinoma, and human breast cancer cells in a time- and dose-dependent manner [26]. However, the impact of MH on hepatocellular carcinoma (HCC) yet remained unknown.

Doxorubicin (DOX) is one of the most effective chemotherapeutic agents that is widely used to treat human malignancies [33]. DOX generates reactive oxygen species (ROS) that provoke the activation of caspases 8 and 3 respectively, thus triggering apoptosis via interaction with Fas-associated protein with death domain (FADD) [34]. Furthermore, DOX blocks DNA synthesis by inhibition of topoisomerase 2b (TOP2B) through intercalation into DNA [35]. Moreover, DOX causes early activation of p53 in tumor cells. Upon activation of the p53 pathway, the expression of the apoptotic regulatory protein, B-cell lymphoma 2 (Bcl-2) is decreased, whereas the downstream caspases, caspase-9, and caspase-3 are activated, leading to apoptosis [36]. However, the clinical use of DOX is limited by its severe side effects and the associated developed drug resistance, thus, single-agent chemotherapy is no longer appropriate for treating human tumors. Recently, combination chemotherapy has emerged as a superior treatment strategy [37].

To the best of our knowledge, the present study represents the first report on the anticancer activity and apoptosis induction of MH and its combination with DOX on HCC cell lines. It is important to investigate the molecular mechanism of the role of MH and combined treatment on HCC cell growth inhibition.

## Results

### MH inhibited HebG2 and Hep3B cell lines proliferation and viability

**Fig. 1:**
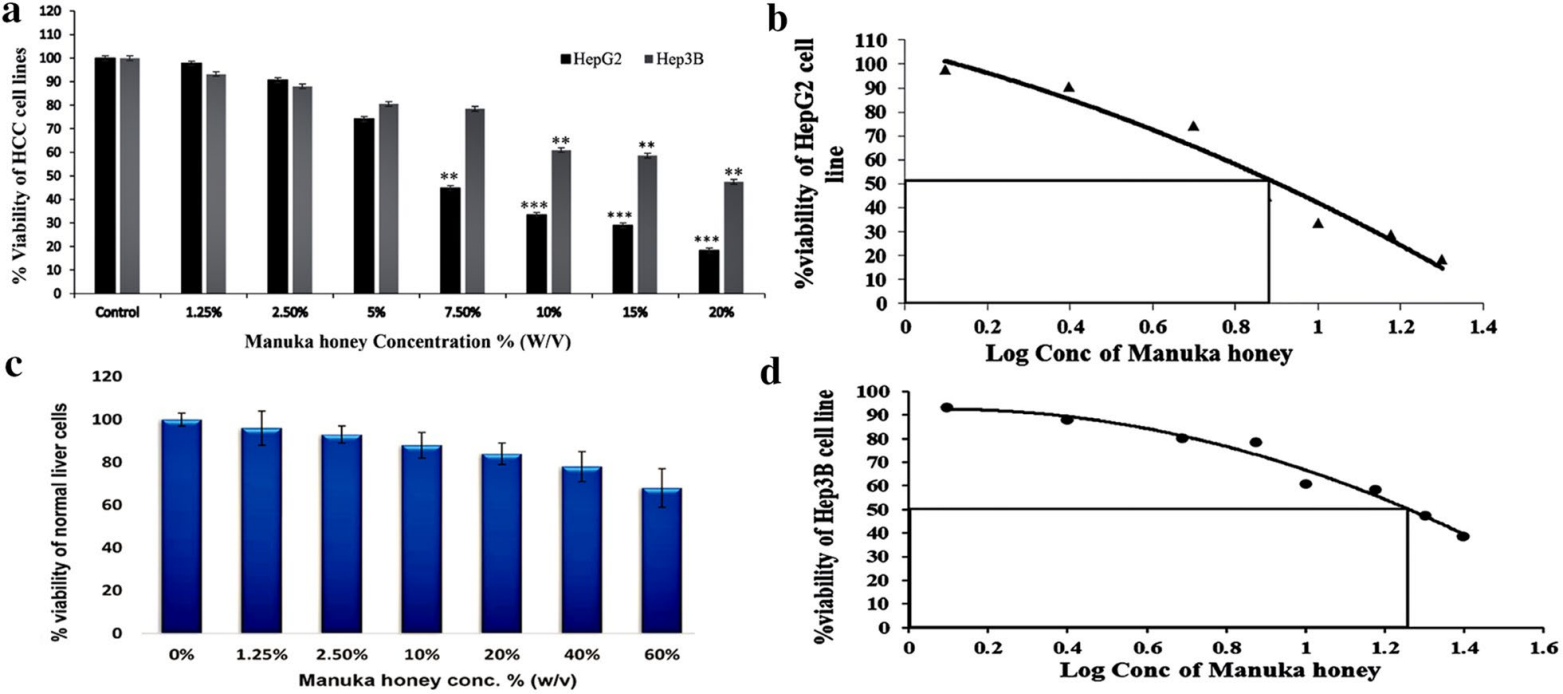
**a** Inhibitory effect of Manuka honey on the viability of HepG2 and Hep3B cell lines: HepG2 and Hep3B cells were grown in a complete DMEM medium and treated with different concentrations of MH (1.25% ~ 20%) for 48 h, and cells' viability was analyzed by MTT assay. Cell viability (%) was compared to control (untreated). Each data point is an average of three independent experiments and expressed as M ± SD. P-value *P < 0.05: **P < 0.01 and *** P < 0.001 compared to untreated control cells. **b**, **d** Dose–response curve of Manuka honey of HepG2 and Hep3B cell lines. **c** Cell viability (%) was compared to control (untreated) showed that MH has no significant cytotoxic effect on the primary normal human neonatal liver cells. The calculated IC_50_ of MH for HepG2 and Hep3B cells was 6.92 ± 0.005% and 18.62 ± 0.07%, respectively. Each data point is an average of three independent experiments and expressed as ± MSD. P-value *P < 0.05: **P < 0.01 and *** P < 0.001 compared to untreated control cells

To determine the cytotoxic effect of MH or combined treatment with DOX on HCC cell lines, the MTT assay was performed on two different HCC cells, HepG2 and Hep3B, respectively. The cytotoxic effects of MH on HepG2 and Hep3B cells were examined by treating the cells with different concentrations of MH (1.25–20%) for 48 h, and the treated cells were normalized to the untreated cells as a control. The MTT assay demonstrated that the treatment of HepG2 or Hep3B cells with increasing concentrations of MH resulted in dramatic cell death and inhibition of cell viability in a dose-dependent manner compared to the control (Fig. 1a). Furthermore, 48 h of MH treatment, the effective significant inhibitory concentrations of MH started at 3.4% and 10% for HepG2 and Hep3B cells, respectively. The concentrations at which 50% maximum inhibition of treated cell viability was achieved and IC_50_ were calculated, using a semi-logarithmic plotting of the percentage of cell viability versus the concentration used for MH. The IC_50_ of MH for HepG2 and Hep3B cells was 6.92 ± 0.005% and 18.62 ± 0.07%, respectively, as shown in (Fig. 1b, d). Combined treatment significantly showed a higher inhibitory effect on cell viability compared to single treatments with MH or DOX in the two tested HCC cell lines. However, HepG2 cells are significantly more sensitive to cell viability inhibition by MH than Hep3B cells. In addition, MH showed no significant cytotoxic effect on the primary human normal neonatal liver cells as shown in (Fig. 1c). In the present study, we focused on the mechanistic inhibitory effect of MH on HepG2 cells that showed a high sensitivity for MH or combined treatment with DOX after 48 h.

**Fig. 2:**
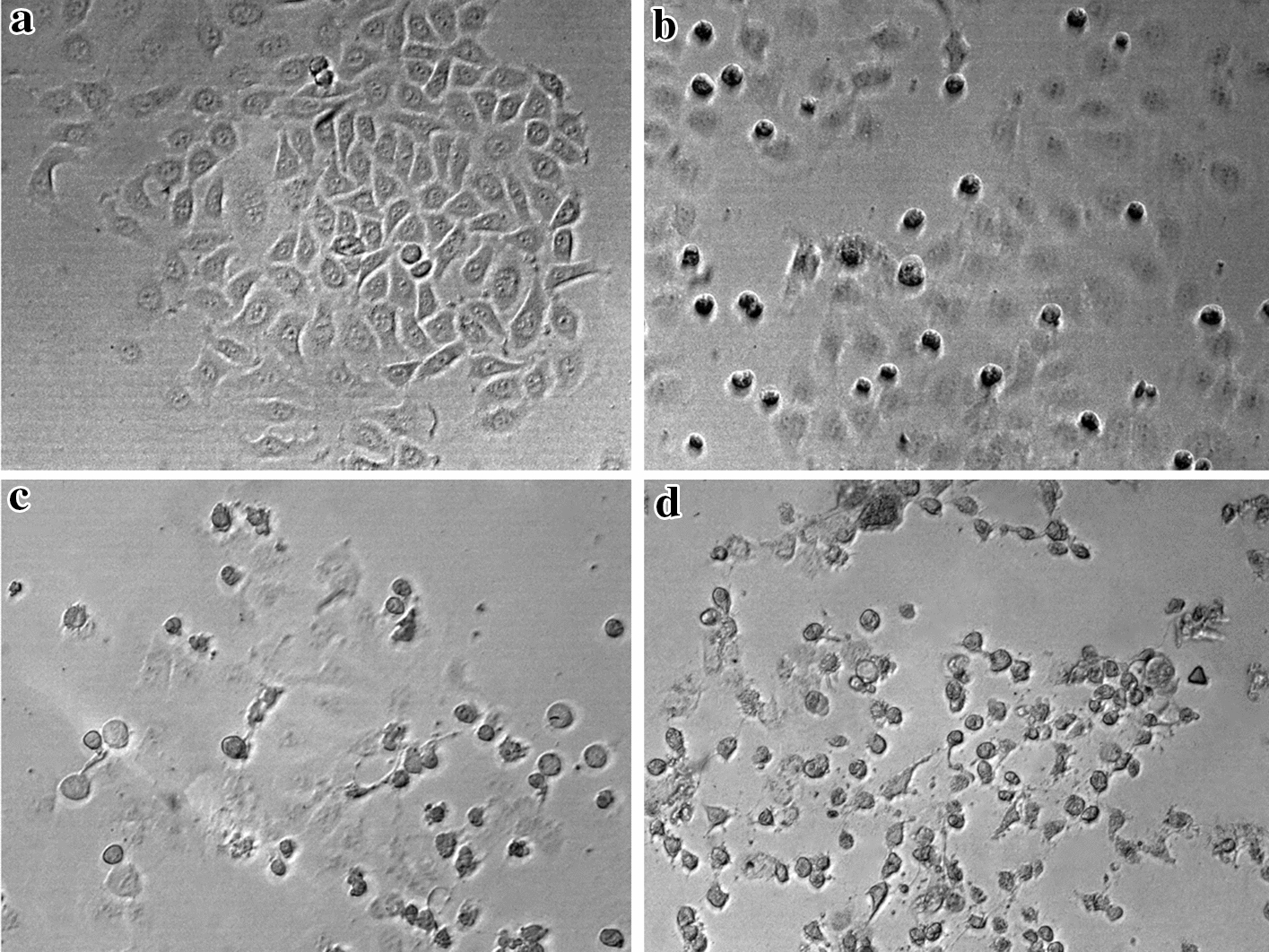
Manuka honey treatment induced morphological changes of HepG2 cells. HepG2 cells were treated with or without as the following: (**a**) Control, (**b**) 1 μM DOX, (**c**) **½** IC_50_ MH, (**d**) **½** IC_50_ MH + 1 μM DOX. Cells were investigated under the inverted microscope at 400X magnification. The above data is representative of three independent experiments

To confirm the data of cell viability and elucidate the effectiveness of MH, or combined treatment on HepG2 cell line, morphological changes of HepG2 cells treated with MH, DOX as a positive control, or ½ IC_50_ MH plus 1 μM DOX combined treatment were studied. The morphological changes of HepG2 were observed by inverted light microscopy as shown in (Fig. 2). After 48 h of treatment, the microscopic examination of HepG2 cells showed dramatic morphological alterations. HepG2 cells became rounded up and displayed a sharp reduction in both number and size. In addition, treated cells showed a complete loss of contact with the neighboring cells, apoptotic bodies, cell shrinkage, detaching from the culture plate, and broken cells compared to their untreated counterparts (Fig. 2). Combined treatment of MH with DOX showed the most dramatic morphological changes compared to treated cells with either MH or DOX. Taken together, MH or combined treatment induced morphological changes with prompted characteristic morphological features of apoptosis in HepG2 cells.

### MH showed a synergistic effect with DOX on caspases activity and apoptosis induction in HepG2 and Hep3B cells

**Fig. 3:**
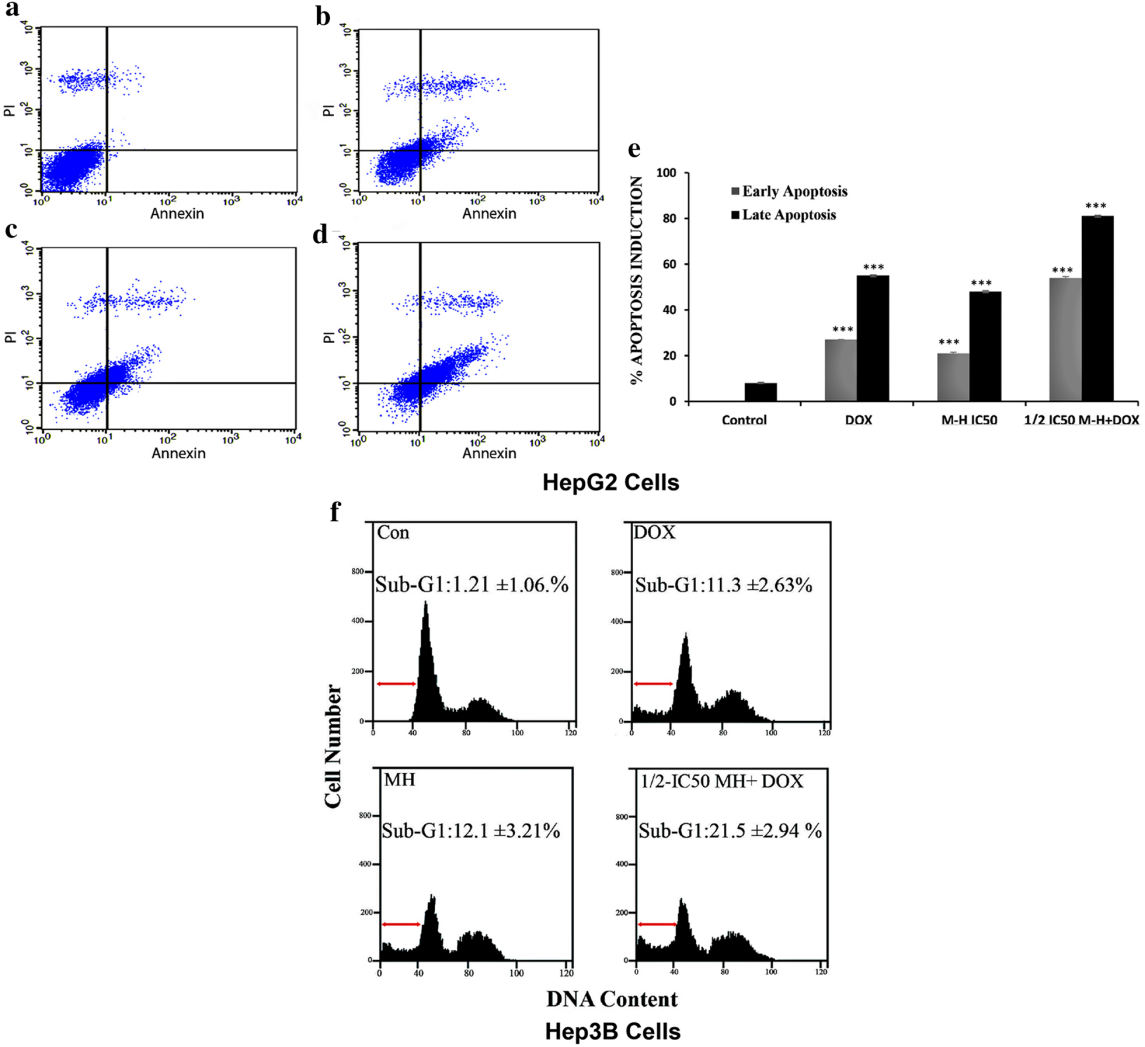
Flow cytometric analysis of early and late apoptosis in treated HepG2 cells with MH, DOX, or combined treatment. HepG2 cells were treated with MH, DOX, or combined treatment for 48 h, stained with FITC-conjugated AV and PI, and subjected to analysis by flow cytometer (**a–d**). The dot plot represents the untreated HepG2 cells as a control (**a**) treated cells with 1 μM DOX (**b**) treated cells with **½** IC_50_ MH (**c**) and cells treated with ½ IC50 MH + 1 μM DOX combined treatment (**d**). The early apoptosis events (AV + /PI−) are shown in, the lower right panels. The late stage of apoptosis (AV + /PI +) is shown in, upper right panels. The bar chart represents the percentage of apoptosis induction in early and late apoptosis in treated HepG2 cells with MH, DOX, or combined treatment. **e** the results are shown as mean M ± SD of three independent experiments, and Statistical analysis was performed using one-way ANOVA with Tukey’s post-hoc test (*P < 0.05, ** P < 0.01, and ***P < 0.001). **f** Cell cycle analysis of Manuka honey induced apoptosis in Hep3B cells. Induction of apoptosis by MH or combined treatment with MH and DOX in Hep3B cells. Hep3B cells were exposed to Control, 1 μM DOX, ½ IC_50_ MH, and ½ IC_50_ MH + 1 μM DOX combined treatment for 48 h. Cell cycle analysis was performed, quantified for treated-Hep3B cells, and (%) sub-G1 was calculated, using flow cytometry as shown by histograms. The results were obtained from three independent experiments. The figures are representative of one of the experimental results

Indeed, apoptosis was quantified and visualized to determine whether the inhibition of cell viability by MH or combined treatment was due to apoptosis induction. [38]. To further investigate the inhibition in HCC cell viability after treatment with ½ IC_50_ and IC_50_ MH, or (½ IC_50_ MH + 1 μM DOX) combined treatment, flow cytometric analysis was performed to examine and confirm the potential mechanism by which MH or combined treatment decreased cell viability. Loss of cell membrane asymmetry, one of the biomarkers of apoptosis, could be detected by Annexin V staining. It represents one of the earliest events in apoptosis (AV+/PI). Therefore, cells that are considered viable (control) are both AV and PI negative (AV−/PI−). On the contrary, the cells staining for both AV and PI (AV+/PI+), mark the late apoptosis stage. Thus, staining with FITC in conjunction with PI dye helps to identify cells in early and late apoptosis stages. HepG2 cells were collected after 48 h treatment with MH, DOX, or combined treatment, then stained with Annexin V-FITC and PI, and finally subjected to flowcytometric analysis. The characteristic dot plots of (Fig. 3a–e) showed that the percentage of early apoptosis remarkably increased by 21%, 27%, and 54% in HepG2 cells treated with MH, DOX, or combined treatment, respectively, compared to the untreated cells (Fig. 3a). Furthermore, there was a significant increase in the percentage of HepG2 cells at the late apoptosis stage by 48%, 55% and 81% after 48 h treatment with MH, DOX, or combined treatment, respectively compared to untreated cells (Fig. 3b, d). In addition, combined treatment induced more dramatic apoptotic events in HepG2 cells compared to either MH or DOX alone, suggesting that MH and DOX act synergistically to inhibit cell proliferation and induced apoptosis in HepG2 cells.

To further elucidate the mechanism of cell growth inhibition, the cell cycle distribution was examined in Hep3B cells after treatment with MH, DOX, or combined treatment for 48 h, and apoptotic cell death was quantified and determined by measuring the cellular DNA content by flow cytometry. After 48 h of treatments, our data showed that the percentage of sub-G1 cells was significantly increased by MH, DOX, or combined treatment, and the apoptotic Hep3B cells (%) in sub-G1 were 12.1 ± 3.21%, 11.3 ± 2.63%, and 21.5 ± 2.94%, respectively, compared to (1.21 ± 1.06%) for control with the highest % of sub-G1 was detected in combined treatment as shown in (Fig. 3f). Overall, these results illustrated that MH or combined treatment has an antiproliferative effect on HepG2 cells via an apoptotic mechanism.

**Fig. 4:**
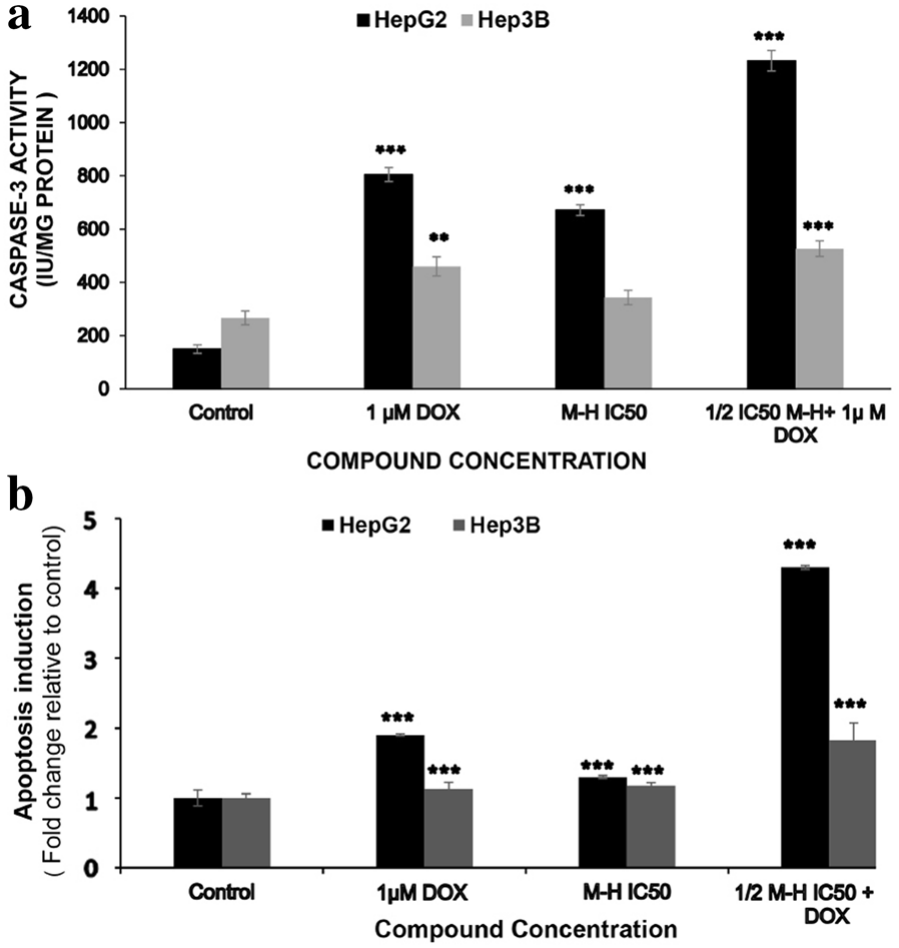
Manuka honey induced Caspase-3 activation and apoptosis induction in HepG2 and Hep3B cell lines. **a** Caspases-3 activity in HepG2 and Hep3B cells. Cells were treated without/with MH, DOX, or combined treatment for 48 h, and caspase-3 enzymatic activity was determined, using a specific colorimetric assay kit according to the manufacturer’s protocol as explained in the Materials and Methods section. **b ** Manuka honey induced Histone-release and apoptotic induction in HepG2 and Hep3B cells. Cells were treated without/with MH, DOX, or combined treatment for 48 h and Histone-release activity was determined, using a specific ELISA assay kit according to the manufacturer’s protocol. Each point is an average of three independent experiments and is expressed as M ± SD. P-value was calculated versus control cells: **P < 0.01 and ***P < 0.001

To examine the regulators that affected the treatment-induced apoptosis in HepG2 or Hep3B cells, caspase-3 activity assays were performed after treatment with MH, DOX, or combined treatment for 48 h. The combined treatment showed a higher increase in caspase-3 activity in both tested cell lines compared to control or individual treatments of MH or DOX, whereas caspase-3 activity was higher in HepG2 cells compared to Hep3B cells in all treated groups as shown in (Fig. 4a, b).

Caspase-3 activity was elevated by 3.5-fold after MH treatment in a dose dependent manner and 4.3-fold with DOX in HepG2 cells. The ½ IC_50_ MH plus 1 μM DOX combined treatment strongly stimulated the activation of caspase-3 in HepG2 cells by a 7.2-fold increase compared to the untreated cells as shown in (Fig. 4).

To further confirm if the reduction in the cell viability was due to apoptosis induction in tested HCC cell lines, an enzyme-linked immunosorbent apoptosis assay was performed, which determines the histone release from apoptotic cells. As shown in (Fig. 4), the treatment of HCC cell lines with MH, DOX, or (½ IC50 MH + 1 μM DOX) combined treatment induced a significant histone release from fragmented DNA compared to the untreated control cells, and the % of apoptosis induction was higher in case of HepG2 than Hep3B cells. Besides, the combined treatment demonstrated a higher degree of apoptosis induction than a single treatment of MH or DOX. In HepG2 cells, the fold increase in the histone-release that results from the DNA fragmentation was elevated by 1.3-, 1.9-, and 4.3-folds for MH, DOX, or combined treatments, respectively, compared to the untreated cells, suggesting that the data of combined treatment of MH and DOX synergistically induced cell death is mainly due to apoptosis in the tested HCC cell lines that was consistent with our flow cytometry results (Fig. 3).

### MH induced apoptosis through modulation of apoptotic signaling-pathways

**Fig. 5:**
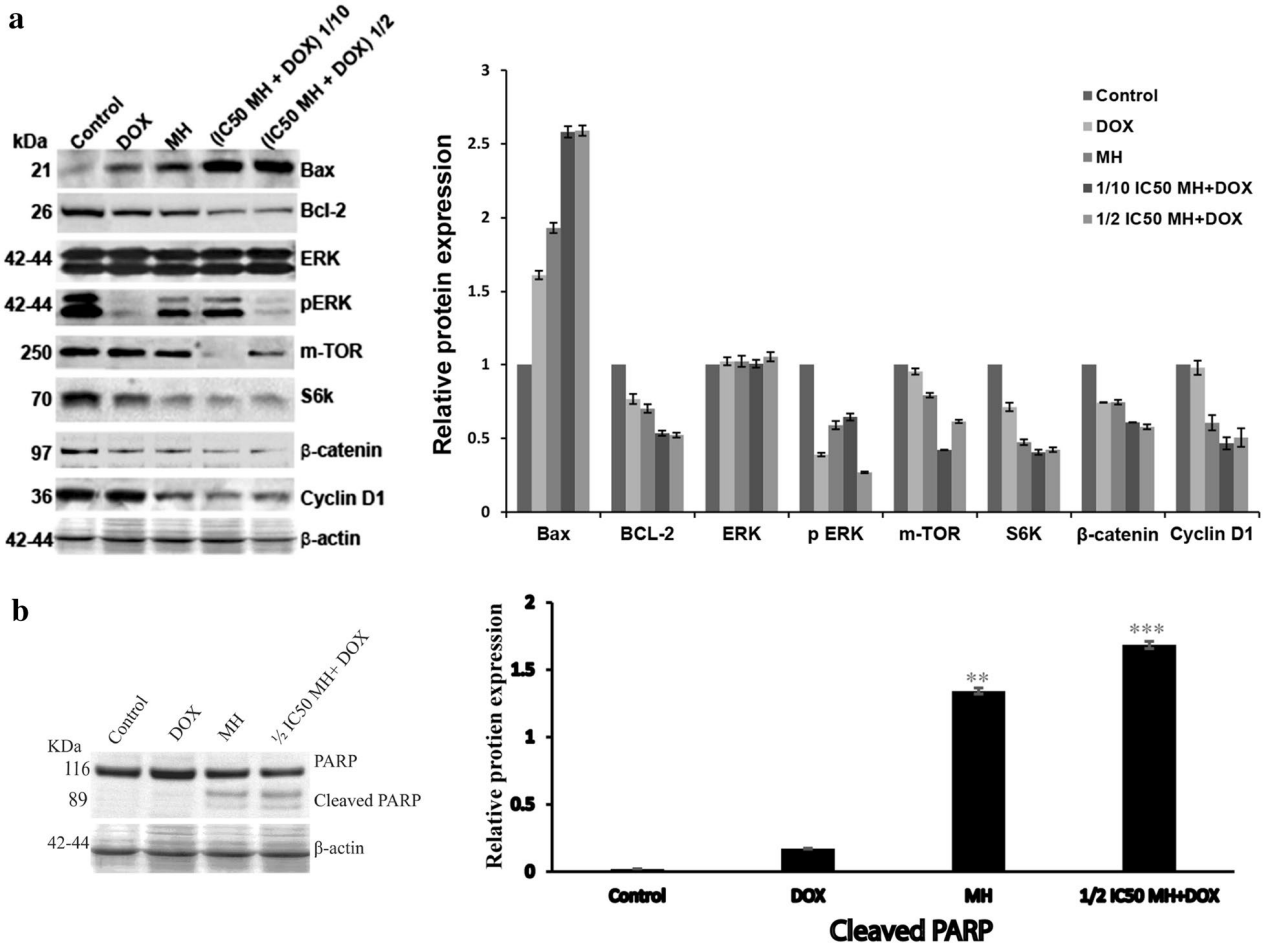
Synergistic effect between MH and DOX modulated signalling pathways that involved in HepG2 cells’ proliferation.** a** (Left upper panel); Protein expression levels of Bax, Bcl-2, ERK, pERK1/2, mTOR, S6K, oncogenic β-catenin, and cyclin D1 were detected by Western blot analysis. **a** (Right upper panel); Relative protein expression levels in HepG2 cells were quantified by Quantity One software. **b** (Left lower panel); Protein expression level of cleaved PARP was detected by Western blot analysis. **b** (Right lower panel); Relative protein expression levels in HepG2 cells were quantified by Quantity One software. HepG2 cells were treated with MH, DOX, or combined treatment for 48 h. The cell lysate was subjected to 10% SDS-PAGE. Proteins were transferred to nitrocellulose membrane and probed with the indicated antibodies. Anti-β-actin was used as a loading control. Protein expressions were quantified normalized to β-actin and controls. Numbers represent the fold of change**.** The experiment was done in triplicate. Data are presented as mean M ± SD. P-value was calculated versus control cells: **P < 0.01 and ***P < 0.001

Western blot analysis was performed for apoptotic markers to explore if the apoptotic induction was due to the activation of intrinsic or extrinsic apoptotic pathways. Many proteins involved in those pathways, were up-or down-regulated, according to their role in the apoptosis pathway. The Pro-apoptotic protein, Bcl-2 associated X protein (Bax), is one of the most important proteins that plays a critical role in the intrinsic apoptosis, and the poly (ADP-Ribose) polymerase (PARP) is a part of the caspase-dependent pathway of apoptosis also, it is cleaved by caspase-3 and leads to apoptosis. Therefore, we investigated the expression level of Bax, Bcl-2, and PARP after MH, DOX, or combined treatments for 48 h. A reduction in Bcl-2 expression and an increase in Bax expression were observed in HepG2 cells after treatment with MH or combined treatment compared to untreated cells. The cleavage expression of Bax and PARP was significantly increased after 48 h of treatment with MH, DOX, or combined treatment, and the highest effect was detected with combined treated cells compared to untreated cells (Fig. 5a). In addition, the most extensive increase in the levels of PARP cleavage was detected at ½ IC50 MH + 1 μM DOX combined treatment, which revealed a 60% increase in PARP cleavage expression (Fig. 5b). Our data revealed that the combined treatment has a more profound effect on upregulation of the proapoptotic gene, Bax, followed by increasing caspase-3 activity, and in turn, PARP cleavage, suggesting a synergetic effect between MH and DOX that was consistent with apoptosis induction and growth inhibition in HepG2 cells.

### Suppression of p-ERK1/2, m-Tor, and β-catenin signaling pathways associated with proliferation and cell survival by MH treatment in HepG2 cells

An extracellular signal-regulated kinase (ERK), mammalian target of rapamycin (mTOR), and β-catenin signaling pathways play an essential and serious role in the pathogenesis of HCC, including cell proliferation and cell survival; moreover, these pathways are up-regulated during the HCC advanced stage [39]. Therefore, the targeting of these pathways gives hope for novel targeted therapies to improve treatment opportunities for HCC. Therefore, we evaluated the protein expression of p-ERK 1/2, mTOR, S6 kinase (S6K), oncogenic β-catenin, and cyclin D1 by western blot analysis. HepG2 cells were treated with MH, DOX, or combined treatment for 48 h. Compared to the control cells, MH or combined treatment significantly downregulated p-Erk1/2, mTOR, S6K, oncogenic β-catenin, and cyclin D1, while no significant effect on the total ERK1/2 was detected. In addition, the most profound reduction in the expression levels of the proteins p-Erk1/2 and oncogenic β-catenin was detected with the ½ IC50 MH + 1 μM DOX combined treatment that showed a 73% and 42% reduction in p-ERK1/2 and oncogenic β-catenin protein expression, respectively, and by 58%, 59% and 53% for mTOR, S6K, and cyclin D1 protein expression as shown in (Fig. 5a). Consistently, our results suggest that MH and DOX act synergistically to inhibit cell viability and induce apoptosis in HepG2 cells.

## Discussion

The present study hypothesized that MH could be a potential candidate and a promising treatment option for HCC cell lines. Our results demonstrated that treatment of MH and particularly its synergetic effect with DOX-induced significant cytotoxicity and apoptosis induction in HCC cell lines, HepG2 and Hep3B, respectively in a concentration-dependent manner. It is worthy to note that Hep2G cells are significantly more sensitive to cell viability inhibition by MH or combined treatment than Hep3B cell line. Both HepG2 and Hep3B were derived from different hepatic progenitor origins and different stages of hepatocyte differentiation which in turn might explain the differences in MH or combined treatment-induced cytotoxicity and apoptosis between Hep3B and HepG2 cells. In addition, MH had no significant cytotoxic effect on the human normal neonatal liver cells. In the present study, we focused on the HepG2 cells that showed a high sensitivity for MH or combined treatment with DOX.

Recently, there is an increased focus directed towards the identification and characterization of novel natural products, such as polyphenols and flavonoids-rich honey [40]. In addition, the new approaches to combined chemotherapies with polyphenols, or polyphenol-containing foods as honey becomes imperative to increase the effectiveness of the chemotherapy, also; this way can overcome the cancer cell resistance and minimize the adverse toxicity. Besides, the mixture of polyphenols existing in whole food is easily consumed and more effective when compared to the single or purified molecule in cancer prevention through synergistic and additive effects [41, 42].

Compared to other honey types, MH has been gaining traction in the research sector, since it is very rich in bioactive compounds such as phenolic and flavonoid compounds [27, 30, 31]. Afrin et al. has reported that MH is enriched with elevated levels of antioxidant compounds, at which the most abundant compounds are flavonols, for instance, quercetin represents 11.81%, luteolin (8.30%), and kaempferol (3.70%) of the total phenolic content [31] and these three compounds have been identified in MH by Alvarez-Suarez et al. [30]. and by Marshall et al. [43] as well. In addition, phenolic compounds such as Gallic acid and syringic acid were the main components in MH, which represents 36.57% and 32.55%, respectively, of the total phenolic content, while the other phenolic acids such as.

4-hydroxybenzoic acid, apigenin, isorhammentin, and caffeic acid were presented in MH in low proportion [31]. MH has been identified with its potential anticancer activity [24]*.* The current study focused mainly on investigating the pro-apoptotic and anti-proliferative effects of MH in HepG2 and Hep3B cell lines. Moreover, our work was carried out, while taking into consideration a priorly conducted study, which demonstrated MH’s safety by showing unaltered hematological and clinical chemistry profiles of MH treated mice [26]. Therefore, the anticancer effect of MH could potentially be specific to HepG2 and Hep3B cells since there is no cytotoxic effect on normal human neonatal liver cells, as opposed to that of the chemotherapy drug, doxorubicin, which was found to be toxic against the normal healthy cells.

Our MTT results proved the cytotoxic effects of MH and combined treatment with DOX on HepG2 and Hep3B cells after treatment with different concentrations of MH (1.25–20%) for 48 h, suggesting that MH is effective in inhibiting HepG2 and Hep3B cells proliferation. Fernandez et al*.* reported on the IC_50_ values of MH on three different cancer cell lines. Firstly, the murine melanoma cells (B16.F1) after 24 h, 48 h, and 72 h of exposure to MH (UMF10+) exhibited IC_50_ values of 2%, 1.3%, and 0.8%, respectively. Similarly, for colorectal carcinoma (CT26) cells, the IC_50_ values recorded at 24 and 72 h, were 2% and 1% respectively. Whereas, the IC_50_ values for human breast cancer (MCF-7) cells, were more than 5% and 4% at 24 and 72 h, respectively [26]. Furthermore, a previous study also demonstrated that the antiproliferative effect of MH was associated with the activation of the caspase-9-dependent apoptotic pathway [26]. Portokalakis et al. reported that MH showed cytotoxicity towards MCF-7 cells after 24 h treatment in a dose-dependent manner, and the concentration of MH, which produced the IC_50_ values was 2.2% for UMF 18 + honey and 4.7% for UMF 5 + honey [44]. In the light of these findings, it has been noted that the IC_50_ values of MH vary among the different types of tested cell lines, according to their molecular, genetic characteristics, and progenitor origins**.** However, differences may also be attributed to the variations in the honey content, especially flavonoid and phenolic acids, which are responsible for the antitumor activities [26, 45]. According to the previous investigations, phenolic compounds such as quercetin, luteolin, kaempferol, gallic acid, and caffeic acid that are the major components in MH, play an important role in the suppression of cancer cell proliferation [26, 41, 45, 46]. On the other hand, according to a previous study, the IC_50_ value of Adriamycin (Doxorubicin) for HepG2 cells was 1.12 μg/ml, which is equivalent to 2 μM [47]. It was previously reported that apigenin, a bioflavonoid present in honey) significantly reversed doxorubicin sensitivity in doxorubicin-resistant hepatocellular carcinoma cell line BEL-7402/ADM and induced caspase-dependent apoptosis [48].

The induction of apoptosis, along with targeting the pathways that regulate and coordinate cellular proliferation and differentiation, is regarded as an important targeted-therapy approach for cancer treatment. Cellular morphological changes are hallmarks of apoptosis and hence microscopic analysis is essential to observe these alterations [11]. Our results showed that the MH treatment of HepG2 cells resulted in apoptotic morphological features. The most pronounced morphological changes occurred at concentrations (½ IC_50_ MH+½ IC_50_ DOX), combined treatment, compared to both the untreated cells and the positive control alone, hence confirming the synergistic effect of the combination treatment. Taken together with the data from the viability assays, this hinted towards the possibility of the potential involvement of an apoptosis-mediated, cellular proliferation inhibitory mechanism. Therefore, to investigate the inhibitory effects of MH or combined treatment, Annexin V assay was coupled with flow cytometry to quantify the MH and DOX treated HepG2 cells, undergoing early and late apoptosis. The Annexin V assay capitalizes on the apoptotic-characteristic early disruption of the plasma membrane and the consequent translocation of the membrane phospholipid, phosphatidylserine to the membrane outer surface. This change in locale facilitates the binding of the phospholipid-binding protein, Annexin V, to phosphatidylserine and thus allowing the detection of the cells undergoing early apoptosis. Whereas, the counterstain PI, is only capable of entering the cells once the integrity of their plasma membranes is greatly diminished, a feature exhibited by the cells undergoing late-stage apoptosis. In the present study, HepG2 or Hep3B cells were treated with MH, DOX, separately, or in combined treatment with MH and DOX for 48 h. MH did not have a significant effect on normal human neonatal liver cells, whereas, in HepG2 and Hep3B cells, the combination treatment synergistically decreased cell viability as seen in (Fig. 1). Furthermore, combined treatment-induced cell death revealed features of apoptosis. The percentage of cells with annexin-V-positive staining and the population of sub-G1 cells, representing apoptosis induction, were also increased in HepG2 and Hep3B, respectively (Fig. 3). Our data showed that the percentage of HepG2 cells undergoing early apoptosis markedly increased to 21%, 27%, and 54% following treatment with MH, DOX, and/or combined treatment, respectively, compared to the untreated cells or single treatment of MH or DOX. Moreover, the percentage of HepG2 cells at the late apoptosis stage exhibited an increase to 48%, 55%, and 81% after 48 h treatment with MH, DOX, or combined treatment, respectively, compared to untreated cells. Our data is in agreement with previous studies, where the mouse melanoma, B16.F1 cells, and human colon cancer, HCT-116 and LoVo, cells exhibited dose-dependent apoptosis induction, upon treatment with MH [47].

The synergistic pro-apoptotic effect attained by using a combination of MH and DOX was evident by the enzyme-linked immunosorbent apoptosis assay on HepG2 cells revealed a significant induction of apoptosis. Hence, we decided to further study this effect on the activity of major apoptotic triggers. Bcl-2 and caspase-3 family members are crucial for the regulation of apoptosis. Moreover, the observed morphological features could be induced by caspase-3 activation that is essential for the characteristic morphological alterations associated with apoptosis [49]. Therefore, we studied the effect of MH on HepG2 cells and focused on caspase-3, a key mediator of the mitochondrial apoptosis events [50]. Upon proteolytic activation by active initiator caspase (caspase-9), caspase-3 proceeds to cleave various substrates, such as poly (ADP-ribose) polymerase (PARP). The aforementioned cleavage of these substrates dictates the characteristic morphological and biochemical features evident in apoptosis [51]. To investigate if MH or the combined treatment would be able to activate caspase-3 in the treated HepG2 and Hep3B cells, caspase-3 activity was assayed. Consistently with our earlier results, MH, or combined treatment showed a significant increase in caspase-3 activity in treated HepG2 and Hep3B cells compared to the untreated cells after 48 h. While the highest increase in caspase-3 activity occurred at concentrations (½ IC50 MH + ½ IC50 DOX) of the combined treatment compared to the single treatment with MH or DOX. The data of HepG2 cells of caspase-3 and apoptosis induction was higher than those of Hep3B cells. Moreover, western blot analysis confirmed the cleavage of PARP and pointed towards the induction of apoptosis, after MH, or combined treatment. All in all, our results suggested that MH, or combined treatment induced apoptosis of HepG2 and Hep3B cells via an extrinsic or an intrinsic apoptosis pathway. Thus, we evaluated whether MH or combined treatment would be able to induce apoptosis in HepG2 cells through the signaling mediated extrinsic apoptosis pathway, or the cell stress-mediated intrinsic apoptosis pathway. In this study, a reduction in Bcl-2 expression and an increase in Bax expression were observed in HepG2 cells after treatment with MH or combined treatment, and the highest induction was detected in combined treatment. Western blot analysis for the pro-apoptotic protein, Bax, was found to be a transcriptional target of p53, a crucial tumor suppressor protein [52]. Moreover, p53, a transcriptional activator, was found to play a role in the induction of the transcription of many genes, including apoptosis-related ones. Therefore, the upregulation of Bax could be triggered via the p53 signaling cascade [52]. Besides, Bax plays an important role in controlling the mitochondrial disruption-mediated cell death, which is marked by the release of cytochrome c into the cytosol [12]. The Bax upregulation coincided with the increase in caspase-3 activity that leads to the activation of PARP cleavage and consequently cell death, suggesting that MH or combined treatment induced apoptosis in HepG2 cells via the activation of the intrinsic (mitochondrial) apoptosis pathway. Consistent with our results, a previous report also showed that MH triggered apoptosis in murine melanoma cells (B16.F1) via the activation of the intrinsic apoptosis pathway [26]. Furthermore, it was reported that the antiproliferative effect of MH was brought about through the activation of the caspase-9 dependent apoptotic pathway, leading in turn to the activation of caspase-3, reduced Bcl-2 expression, DNA fragmentation, and finally cell death [26]. Moreover, another study demonstrated that Chrysin, a common flavonoid of MH, induced apoptosis via the activation of the p53/Bcl-2/caspase-9 pathway in HCC (HepG2) cells [53]. Furthermore, Im et al. reported that luteolin (the major phenolic compound of MH) activated caspase-8, − 9, and − 3, and cleaved PARP in human hepatocellular carcinoma SK-Hep-1 cells [54]. Besides, it was reported that quercetin (common flavonoid of MH) remarkably inhibited HCC cell (HepG2 and SMCC-7721) proliferation and induced apoptosis by upregulating the expression of Bad and Bax and downregulating the expression of Bcl-2 in vitro [55].

RAF/MAPK/ERK signaling pathway is activated in HCC and was found to correlate with the advanced stage of the disease [39]. Therefore, we evaluated the potential effect of MH on the RAS/ERK pathway by studying the protein expression of total ERK1/2 and their phosphorylation by western blot. The phosphorylation of ERK1/2 is essential for their activation, and hence studying their phosphorylation and relating it to the total protein abundance, sheds light on the potential effect of MH or combined treatment on ERK1/2 activity. The treatment of HepG2 cells with MH, or combined treatment for 48 h, elicited a significant decrease in the p-ERK1/2, despite the total ERK1/2 abundance exhibiting no marked changes. In addition, the most profound reduction in p-ERK1/2 and was recorded with the combined treatment of (½ IC_50_ MH + 1 μM DOX), which showed a 73% reduction in the p-ERK1/2, reaffirming the synergistic effect of the combination employed, suggesting the anticancer and antiproliferative effect of MH might be mediated through the RAS/ERK signaling pathway*.* Complementing our data, a priorly conducted study has revealed that Gelam honey alone and /or combined with ginger, induced early apoptosis, partly through the RAS/ERK pathway, in colorectal cancer cells [56]. Furthermore, Ding et al. demonstrated that quercetin reduced cell viability, induced apoptosis, cleaved caspase-3, cleaved PARP, upregulated Bax protein expression, and downregulated p-ERK1/2 protein levels in HepG2 cells [57]. Shao et al. demonstrated that phytochemicals, such as apigenin (one of the flavonoids present in MH), downregulates the activation of ERK and Akt in human colon cancer cells [58]. Moreover, ERK1/2 has been previously implicated with β-catenin, mTOR, and cyclin D1 in cellular proliferation [39, 59]. Hence, studying the abundance of these proteins following the treatment of HepG2 cells with MH should help to better understand the underlying molecular mechanisms associated with the anti-proliferative and pro-apoptotic effect of the honey.

mTOR and its downstream effector, S6K, are important regulators of protein translation. Moreover, the mTOR signaling pathway was found to upregulated in several carcinomas, including HCC, where overexpression of S6K was recorded in nearly half of the studied HCCs [60]. Furthermore, the Wnt/β-catenin pathway (Canonical Wnt signaling) has been found to enhance the m-TOR-mediated modulation of S6K [61]. The crosstalk between the RAS/ERK and mTOR signaling pathways to positively and negatively regulate one another is well documented, while the simultaneous inhibition of both pathways induced an anti-proliferative effect on different cancer cells [62]. The protein expression of both mTOR and S6K in HepG2 cells following treatment with MH, DOX, or combinations of MH and Dox showed inhibition in cell proliferation, where both proteins exhibited a significant downregulation, with the most marked inhibition being evident in the cells treated with combinations of MH and DOX. The present results may be attributed to quercetin (one of the major phenolic compounds of MH). Wu et al. reported that quercetin inhibited the proliferation of HCC cells via the downregulation of hexokinase-2 (HK2) protein level and suppression of the AKT/mTOR pathway [63]. In addition, Ji et al. have demonstrated that quercetin induced autophagy via inhibiting the AKT/mTOR pathway in HCC [64]. Another study reported that luteolin reduced cell viability and induced apoptosis in prostate cancer cells, besides, it downregulated AKT, ERK, mTOR, and P70S6K [65]. Luteolin downregulated the protein expression levels of phosphorylated Akt, mTOR, p70S6K, and MAPK, also induced caspase and PARP cleavages in glioblastoma cells and promoting cell cycle arrest [66].

Moreover, we further studied the effect of MH on the oncogenic β-catenin expression, a transcription factor, which plays a critical role in HCC progression. β-catenin is a multifaceted protein, as it plays an integral role in the maintenance of the E-cadherin-catenin cell adhesion complex in the cellular junctions, through mediating the binding of cadherins to the actin cytoskeleton [67]. In addition, β-catenin is also a key protein in the canonical Wnt signaling pathway [68]. The activation of this pathway induces the cytoplasmic accumulation of β-catenin and its consequent translocation into the nucleus, thus triggering the transcription of downstream target genes [69]. The Abnormal upregulation of the Wnt/β-catenin signaling pathway is the hallmark of many tumors, including liver cancers, where the upregulation of β-catenin plays an important role in the development and progression of HCC [68]. Therefore, various approaches have been conceived and executed throughout the past years to target the Wnt/β-catenin pathway, as a means to develop novel therapies for HCC treatment [70]. The treatment of HepG2 cells with MH or combined treatment resulted in a significant reduction in the abundance of β-catenin, with the most profound decrease being recorded under the combined treatment, comprising both MH and DOX.

In the light of our western blot results, we assumed that there is a connection between the decreased p-ERK1/2 and the decrease in β-catenin expression. This inhibition pattern of β-catenin expression correlates with the decreased p-ERK1/2, implying that MH may be down-regulating β-catenin through the Ras/ERK signaling pathway. Benn et al. revealed that around 50–70% of HCC patients exhibited increased p-ERK due to the activated RAS/ERK pathway. The activation of this pathway is associated with the inhibitory phosphorylation of Glycogen synthase kinase 3 beta (GSK-3β), which acts as a negative regulator of the canonical Wnt signaling, resulting in the inactivation of GSK-3β and the accumulation of β-catenin [71]. The (½ IC_50_ MH plus 1 μM DOX) combined treatment markedly reduced the protein expression levels of β–catenin in HepG2 cells after 48 h, effectively validating the synergistic effect of the combined treatment MH and DOX. Consistent with our results, It was uncovered that MH possesses components, such as polyphenols and quercetin flavonoids, which were found to contribute to its antiproliferative properties, through reducing β-catenin/Tcf transcriptional activity and down-regulating the canonical Wnt signaling pathway in colon cancer cells [63]. Moreover, the MH bioflavonoid Galangin was found to diminish the β-catenin response transcription (CRT), which is abnormally elevated in colorectal and liver cancers [72]. This bioflavonoid acts by enhancing the degradation of the intracellular β-catenin, as well as restraining the β-catenin/T-cell factor-dependent gene expressions, such as cyclin D1 and c-myc. Thus, it consequently exerts an anti-proliferative effect on the CRT-positive cancer cells [72]. In conclusion, the phytochemicals present in MH suppressed β-catenin in HepG2 cells, thus contributing to the MH’s anti-cancer effect [73].

Previous studies demonstrated that the overexpression of cyclin D1 could be attributed to β-catenin activation and the RAS/ERK signaling pathway [74, 75]. Cyclin D, a crucial regulator of the cell cycle progression, is overexpressed in different cancers, especially in HCC [76]. Moreover, it is a downstream target of β-catenin and RAS/ERK signaling pathway in HCC, where the increased p-ERK1/2, results in elevated expression of cyclin D1 and the inhibitory phosphorylation of the tumor suppressor protein, pRb [77]. Besides, the overexpression of cyclin D1 was found to be involved in HCC tumor cell differentiation, therefore targeting cyclin D1, is regarded as an alternative approach in cancer therapy [78]. In the present study, the cyclin D1 expression in MH treated HepG2 cells, revealed a significant downregulation with the most significant decrease being exhibited by the cells treated with combined treatment. Our data demonstrated that cyclin D1 might be one of the downstream targets of β-catenin and ERK1/2 in HepG2. Consistent with our results, MH contains high amounts of quercetin, which can profoundly inhibit the growth and proliferation of HepG2 cells by reducing the expression of cyclin D1 [79].

## Conclusion

**Fig. 6:**
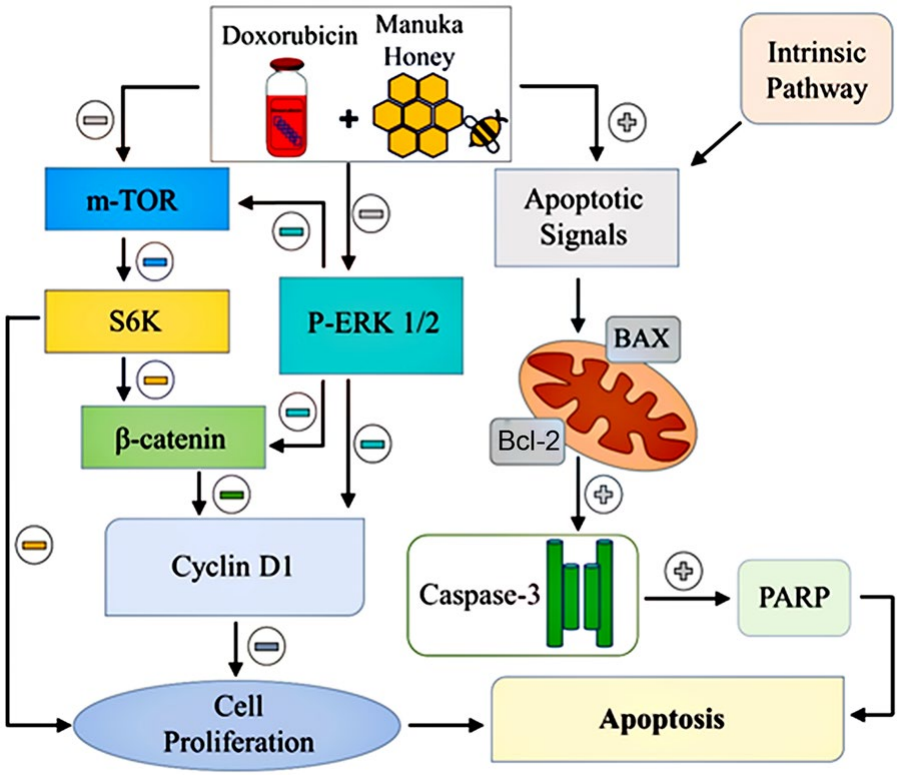
Proposed schematic overview of apoptosis induced by MH via modulation of signaling pathways that involved in HCC proliferation and apoptotic markers in human HepG2 cell line

All in all, to our knowledge, the present study provides the first report on the anticancer activity of MH on HCC cells. Moreover, combined treatment of MH and DOX showed a potent therapeutic effect more than the individual treatment through inhibition of several oncogenic signal transduction proteins oncogenic β-catenin, pERK1/2, mTOR, S6K, and cyclin D1. Furthermore, we uncovered a potential correlation between p-ERK1/2, β-catenin, S6K, and cyclin D1, which might be responsible for the anti-cancer effect of MH or combined treatment. We also demonstrated the possibility of apoptosis induction via Bax upregulation and down-regulation of Bcl-2, followed by the activation of caspase-3 that caused an elevation in DNA fragmentation, leading to increased histone release, increased PARP cleavage, and finally cell death. Hence, a possible molecular mechanism, underlying the observed antiproliferative and proapoptotic activity of MH or the combined treatment of MH and DOX in HCC progression was illustrated as shown in (Fig. 6). Taken together, the present study serves to shed light on the anticancer mechanism of MH and its synergistic inhibitory effect on DOX-mediated apoptotic cell death in HCC cell lines, while introducing the naturally occurring, MH, as a promising antitumor and non-toxic candidate with possible potential effective adjuvant therapeutic for HCC treatment.

## Material and methods

### Cell line

HCC Hep3B and HepG2 cells were obtained from the American Type Culture Collection (Manassas, VA, USA). Cells were maintained in Dulbecco's modified Eagle's medium (DMEM) supplemented with 10% (v/v) fetal bovine serum (FBS), (100 IU/ml) penicillin/streptomycin, and (50 IU/ml) Fungizone (all reagents were produced by Lonza Bio-Whittaker, Walkersville, USA). The cultures were maintained at 37 °C in 5% CO_2_ atmospheric humidity (Laminar Air Flow Cabinet “Safety Cabinet” type II & CO_2_ Incubator, Shell, USA). Primary human normal neonatal liver cultures (PHNL) were established at Georgetown University, using the conditional reprogramming (CR) of the cell method as previously described [80]. All collected tissue samples and the research protocols were performed in accordance with the Institutional Review Board and approved by the ethical committee of GU Hospital and GU Medical Center, USA (Protocol’s No. MOD00001211). Mycoplasma detection assay was performed by Lombardi Tissue Culture & Bio-banking Shared Resource (TCBSR,) using MycoAlert detection kit (cat #LT-07118, Lonza Nottingham, LTD). The PHNL-CR cells were carried in culture for over 30 passages. All comparative studies were performed, using the earliest and most comparable passages available.

### Drug and compounds

Manuka honey (MH) (UMF 10 + , Ultra-pure honey for a medical grade, Advancis Medical, Nottingham, UK) was diluted to different concentrations (% w/v) in culture medium for use. All preparations of MH were freshly prepared on the day of use. Doxorubicin (DOX) was purchased from Sigma Aldrich (St. Louis, MO, USA) and before each experiment, the drug was diluted to the required final concentration in the culture medium.

### MTT cell proliferation assay

HepG2 and Hep3B cells were cultured till mid-log phase in 100 µL of medium and seeded in a 96-well plate at a density of 1 × 10^4^ cells/well for 24 h before treatment. The seeding media was then changed to media with different concentrations (1.25% to 20%) of MH (untreated cells were used as the negative control) for 48 h. Then 10 µL of the 12 mM MTT stock solution (Invitrogen Corporation, CA, USA) was added to each well. After incubation for 4 h at 37 °C, 100 µL of the prepared SDS-HCL solution (10 ml of 0.01 M HCL was added to one tube containing 1 g SDS mixed gently by sonication) were added to each well and mixed thoroughly, using the pipette. The microplate was incubated at 37 °C for 18 h in a CO_2_ incubator. The absorbance was then measured at 545 nm using a microplate ELISA reader (Enzyme-linked immunosorbent assay reader, ELISA Unit, Stat Fax 2100, USA) and IC_50_ was determined. To achieve significant quantitative analysis, experiments were repeated in triplicate in parallel for each concentration and compared to untreated control experiments.

### Cell morphology

Equal numbers of HepG2 cells/well in supplemented DMEM medium were seeded in 12-well culture plates. The cells were treated with ½ IC_50_ MH, 1 μM Doxorubicin, as a positive control, or ½ IC_50_ MH plus 1 μM DOX as a combined treatment. The cells were incubated in 5% CO_2_ at 37 °C for 48 h, washed with cold PBS (1X), and fixed with 10% buffered formalin [81]. Cells were examined by inverted microscopy at 400 × magnification (Inverted Microscope, Optika, Italy). Digital images were acquired with a Kodak microscopic digital camera.

### Flow cytometry analysis of apoptosis

To determine apoptotic cell death, annexin-V-positive cells and the ratio of sub-G1 were analyzed by flow cytometry (BD Sciences, Franklin Lakes, NJ, USA). Annexin-V (AV) assay was performed to quantify early and late apoptosis in HepG2 cells treated with IC_50_ MH, 1 μM DOX, or ½ IC_50_ MH + 1 μM DOX combined treatment compared to untreated cells. This assay was performed by flow cytometry, utilizing Annexin V-FITC/PI apoptosis detection kit according to the manufacturer’s protocol (Becton Dickinson, CA, USA). Briefly, HepG2 cells were seeded at a density of 2 × 10^5^ in a 6-well plate, treated with IC_50_ MH, 1 μM DOX, or ½ IC_50_ MH + 1 μM DOX combined treatment and incubated for 48 h. Cells were collected and stained with AV-fluorescein isothiocyanate (FITC) and Propidium iodide (PI), and quadrant statistics were used to identify early and late apoptotic cells. To measure the sub-G1 DNA population and apoptotic cell death of Hep3B cells, cells were stained by PI solution and FITC annexin-V, respectively, and analyzed with the flow cytometer.

### Caspase-3 activity

The caspase 3 colorimetric assay is based on the hydrolysis of the peptide substrate acetyl-Asp-Glu-ValAsp p-nitroanilide (Ac-DEVD-pNA) by caspase 3, resulting in the release of the p-nitroaniline (pNA) moiety. p-Nitroaniline has a high absorbance at 405 nm. The concentration of the pNA released from the substrate is calculated from the absorbance values at 405 nm or from a calibration curve prepared with defined pNA solutions. Caspase-3 activity was assayed, according to the manufacturer’s protocol of colorimetric assay kit (Bio Vision, Inc., CA, USA) that provides quick and efficient detection of caspase-3 activity in cell lysates and purified preparations of caspase-3. 5 × 10 ^6^ cells were treated with MH, DOX or (½ IC50 MH + 1 μM DOX) combined treatment and lysed in 100µL lysis buffer containing 10 mM HEPES (4-(2-hydroxyethyl)-1-piperazine ethane sulfonic acid), pH 7.4, 2 mM EDTA, 0.1% CHAPS (3-[(3-cholamidoprpyl) dimethyl ammonio]-1-prpropane sulfsulfonic acidacid0µg/mL PMSF (phenyl methane sulfonyl fluoride or phenylmethylsulfonylfluoride), and 5 mM DTT (Dithiothreitol). Cells were homogenized by three cycles of freezing and thawing and then centrifuged to remove the cellular debris. Each sample was then incubated in buffer containing 10 mM HEPES, pH 7.4, 2 mM EDTA, 0.1% CHAPS, and 5 mM EDTA supplemented with its substrate (AcAsp-Glu-Val-Asp-AFC) Ac-DEVD-AFC for 1 h at room temperature and the reaction was stopped with 1 N HCl. Optical density was measured using a spectrophotometer at 405 nm (Jenway Spectrophotometer, UK). The data are expressed as relative fold increase compared to control (non-treated cells). Each assay was done in triplicate and the standard error of the mean was determined.

### Apoptosis induction assay (ELISA)

An ELISA assay was performed, using Cell Death Detection ELISA PLUS Kit (Roche-Applied Science, Indianapolis, USA) that measures histone release from fragmented DNA in apoptotic cells. Firstly, cells were seeded (2 × 10^4^) in a 96-well plate for 24 h, then incubated for 48 h in media contain MH, DOX, or combined treatment. Cells were lysed with 200 μL lysis buffer for 30 min at 4 °C, the lysates were subjected to centrifugation for 10 min then, and 20 μl of the collected supernatant was incubated with anti-histone biotin and anti-DNA peroxidase for 2 h at room temperature. After three washes with the incubation buffer, 100 μl of substrate solution (2,2′-azinodi (3-ethylbenzthiazoline-sulphuric acid)) was added to each well and incubated for 15 min at room temperature followed by the addition of 100 μl ABTS stop solution. Finally, the absorbance was measured using an ELISA reader (Jenway Spectrophotometer, UK) at 405 nm. Each assay was performed in triplicate and the standard error of the mean was determined.

### Western blot analysis

HepG2 cells were plated in 25 cm^2^ flask and incubated under cell culture conditions. (37 °C, 5% CO_2_). On the following day, cells were treated with fresh medium containing MH, DOX, or ($${\raise0.5ex\hbox{$\scriptstyle 1$} \kern-0.1em/\kern-0.15em \lower0.25ex\hbox{$\scriptstyle {10}$}}$$ IC50 MH+1µM DOX and ½ IC_50_ MH+1 µM DOX) combined treatment. After 48 h, cells were harvested, washed in phosphate-buffered saline (PBS), and lysed in lysis buffer [250 mM NaCl, 25 mM Tris–Cl (pH 7.5), 5 mM EDTA (pH 8.0), 1% NP-40, 1 mM 4-(2-aminoethyl) benzenesulfonyl fluoride hydrochloride, 5 mM dithiothreitol, and protease inhibitor cocktail] followed by centrifugation at 15,000 r.p.m for 30 min at 4 °C. The supernatants were collected, and the protein concentrations of the collected lysates were determined, using the BCA protein assay kit (Thermo Fisher Scientific Inc., Rockford, IL, USA). Samples were prepared to be loaded on the gel by adding 2-mercaptoethanol (Fluka Sigma-Aldrich, St. Louis, Missouri, USA), distilled water, and 4X Laemmli buffer as a loading buffer then denatured by boiling at 98 °C for 5 min. 20 µg/µL of total protein were loaded per mini-gel well and separated on 10% Tris-SDS-PAGE gels (Sodium Dodecyl Sulphate Polyacrylamide Gel Electrophoresis) (Vertical Electrophoresis Unit, Bio-Rad, Laboratories, USA) at 90–120 V at 2 h. The separated proteins were transferred to UltraCruz™ nitrocellulose pure transfer membrane (Santa Cruz Biotechnology, CA, USA), using the protein blotting device (Bio-Rad, Laboratories, USA) containing transfer buffer. The membrane was blocked with 3% BSA solution in TBST buffer (TBS 1X (10 mM Tris HCl, pH 7.4, 150 mM NaCl) and 0.1% Tween 20) for 1 h under agitation. After blocking, the membrane was incubated overnight with the primary antibody at 4 °C as the following: anti-β-catenin, anti-cyclin D1, anti-total ERK1/2, anti-pERK1/2, anti-mTOR, anti-S6K, anti-PARP, anti-Bcl-2, and anti-Bax, (1:1000; Santa Cruz Biotechnology, CA, USA). The expression of the β-actin protein (Sigma-Aldrich, St. Louis, Missouri, USA) was used as a loading control for protein normalization. Following incubation with the primary antibodies, the membrane was washed three times in TBST with agitation. The membrane was subsequently probed with the appropriate horseradish peroxidase (HRP-) conjugated secondary antibody (anti-mouse or antirabbit) (1:5000; Santa Cruz Biotechnology, CA, USA) for 1 h at room temperature with agitation followed by three washes with TBST buffer, and finally, the membranes were developed by an enhanced chemiluminescent reagent (Amersham Biosciences, Westborough, MA, USA), and then photographed. Densitometry of the developed bands was performed, using Quantity One Analysis Software (Bio-Rad Laboratories, USA). Membranes were stripped using blot stripping buffer (Thermo Scientific, lL, USA) and re-probed with anti-β-actin as a control for equal loading.

## Statistical analysis

The results were presented as the mean ± SDM of at least three independent experiments. A one-way analysis of variance (ANOVA) was performed using SPSS-IBM software, version 20 (SPSS Inc., Chicago, IL, USA) followed by Tukey’s honestly significant difference (HSD) post hoc test (P < 0.05). *P* vaP-value calculated versus control cells: *P-value < 0.05; **P < 0.01 and ***P < 0.001 were considered statistically significant.

## Data Availability

The datasets used and/or analyzed during the current study are available from the corresponding author on reasonable request.

## Abbreviations

Akt: Protein kinase B
AV: Annexin-V
Bcl-2: B-cell lymphoma-2
Bax: Bcl-2 associated X protein
cyclin D1: Cyclin-dependent protein kinase activity in G1 of the cell cycle
DOX: Doxorubicin
DMEM: Dulbecco's modified Eagle's medium
Erk1/2: Extracellular signal-regulated kinase ½
FBS: Fetal bovine serum
FITC: Fluorescein isothiocyanate
HCC: Hepatocellular carcinoma
HPLC: High-performance liquid chromatography
HEPES: 4-(2-Hydroxyethyl)-1-piperazine ethane sulfonic acid
h: Hour
IC50: The half maximal inhibitory concentration
MH: Manuka honey
mTOR: Mammalian target of rapamycin
PARP: Poly (ADP-ribose) polymerase
PBS: Phosphate-buffered saline
PI: Propidium iodide
ROS: Reactive oxygen species
S6K: Ribosomal protein S6 kinase
SDS-PAGE: Sodium Dodecyl Sulphate Polyacrylamide Gel Electrophoresis
TOP2B: Topoisomerase 2b
TBST: Tris-buffered saline containing Tween-20
r.p.m: Round per minute
UMF: Unique manuka factor
